# Printed Wide-Slot Antenna Design with Bandwidth and Gain Enhancement on Low-Cost Substrate

**DOI:** 10.1155/2014/804068

**Published:** 2014-02-13

**Authors:** M. Samsuzzaman, M. T. Islam, J. S. Mandeep, N. Misran

**Affiliations:** Department of Electrical Electronic and Systems Engineering, Faculty of Engineering and Built Environment, Universiti Kebangsaan, 43600 UKM Bangi, Selangor D.E, Malaysia

## Abstract

This paper presents a printed wide-slot antenna design and prototyping on available low-cost polymer resin composite material fed by a microstrip line with a rotated square slot for bandwidth enhancement and defected ground structure for gain enhancement. An I-shaped microstrip line is used to excite the square slot. The rotated square slot is embedded in the middle of the ground plane, and its diagonal points are implanted in the middle of the strip line and ground plane. To increase the gain, four L-shaped slots are etched in the ground plane. The measured results show that the proposed structure retains a wide impedance bandwidth of 88.07%, which is 20% better than the reference antenna. The average gain is also increased, which is about 4.17 dBi with a stable radiation pattern in the entire operating band. Moreover, radiation efficiency, input impedance, current distribution, axial ratio, and parametric studies of S11 for different design parameters are also investigated using the finite element method-based simulation software HFSS.

## 1. Introduction

In modern wireless communication systems, the demand for wide and multiband antennas is increasing to support multiusers and to provide more information with higher data transmitting and receiving rates. Among different kinds of antennas, microstrip antennas are one of the most prominent structures due to their light weight, compatibility, low profile, ease of fabrication, multifrequency capability, and low cost. Compared with the three conventional types of antennas, planar microstrip antennas on small pieces of printed circuit board (PCB) have become familiar in recent wireless communication, because they can be easily embedded into wireless devices or integrated with other radio frequency (RF) circuitry. Generally, a planar structure can be used to minimize the volumetric dimension of a wide band antenna by replacing three-dimensional radiation elements with their planar design [1].

Various types of antennas have already been designed for wideband and multiband applications. Various dielectric materials have been used for designing and prototyping these antennas. Basically, a dielectric material chosen for the design of wideband antennas is preferable to feature a higher permittivity and lower dissipation factor [2]. Materials with higher dielectric constant have a higher capability of storing charge and produce larger electromagnetic fields. However, they have limited isolation between conductors. On the other hand, materials with lower permittivity are good insulators for lower-frequency signals that require high isolation in densely packed circuits such as mobile and satellite communications [3]. The search for the latest improved materials that could be used in place of aluminum or other conductors is an important task for many applications in the wireless industry. Size and weight reduction, more tolerance to fatigue, and ease of manufacturing for complex structures are some advantages of using such materials. Such kinds of characteristics can lead to operational cost reduction and performance intensification. The woven glass fabric with epoxy resin (FR4) composite is a successful and popular example of such materials in many applications. Several studies have been undertaken in recent years to explore the performance of this material for antenna development. Moreover, by using a material with higher permittivity a compact antenna that is capable of achieving very wide or multioperating band can be designed [4–7]. A ceramic-polytetrafluoroethylene (PTFE) composite material-based miniaturized split-ring multiband patch antenna was designed [4]. The proposed antenna obtained operating bandwidths (reflection coefficient <−10 dB) ranging from 5.0 to 6.5 GHz (1.5 GHz), 9.1 to 9.6 GHz (500 MHz), and 10.7 to 11 GHz (300 MHz). However, the antenna was designed on high dialect and costly substrate. A miniaturized modified circular patch antenna was designed on ceramic-PTFE composite material with dimensions 0.22*λ* × 0.29*λ* × 0.23*λ*; the proposed antenna achieved multi-band characteristics. However, the antenna failed to fulfil the requirement of Wi-Fi/WiMAX applications [5]. A compact square loop multiband patch antenna design on high dielectric ceramic composite material was proposed [8]. Although the reported antenna achieved multiband, impedance bandwidth was low and substrate cost was high compared with epoxy resin fibre. A wideband pentagon-shaped microstrip slot antenna was designed on epoxy resin composite material [9]. The proposed antenna design obtained 124% impedance bandwidth, but its use in portable communication devices was limited due to the large ground plane. A double *L*-shaped multiband patch antenna on polymer resin substrate material was designed [10]. The proposed design achieved dual operating band centred at 4.85 GHz and 8.1 GHz, which failed to cover WLAN/WiMAX applications. As is well known, antennas with various shapes, such as circle [11], ellipse [12], and triangle [13] CPW fed [14, 15], rectangular patch with partial ground plane [16], have been stated to have wide bandwidth. Each slot shape requires a feed stub of appropriate shape. An optimum impedance bandwidth can be obtained by the coupling between the feeding structure and the slot [17–19]. A printed wide-slot antenna [17] fed by a microstrip line with a fork-like tuning stub provided broad bandwidth through the proper parameters of the fork-like tuning stub. It was reported in [18] that introducing an *L*-shaped slot with a *W*-shaped feed stub can improve bandwidth. The authors in [20, 21] proposed a novel bandwidth enhancement technique for a microstrip-fed wide-slot antenna based on fractal shapes. By etching a wide slot as fractal shapes, the bandwidth of the proposed wide-slot antenna was significantly enhanced. However, it made the configuration of the wide-slot antenna more complicated. The square slot antenna has a relatively wider bandwidth than other types of antennas, but its applicability as a broadband antenna is limited due to the characteristics of a single resonant mode. In [22], by rotating the square slot, the other resonant mode operating near one of the conventional wide-slot antennas could be obtained. As a result, a wide operating bandwidth of about 2.2 GHz (49.4%) with respect to the centre frequency at 4.453 GHz was obtained. However, it is not enough for the operating bandwidth to cover more wireless communication services. There is still room to explore miniature antennas with wideband, high gain, and more efficiency with different material substrate.

In this paper, a novel rotated square slot position in defected ground structure is used to obtain wideband characteristics with gain enhancement. The proposed rotated square slot diagonal point embedded in the middle of the patch and strip line and defected ground structure consists of four *L* slots. A detailed simulation is conducted to understand the antenna behaviour and optimize the square slot diagonal position and *L* slots for broadband operation. Finally, the proposed design is implemented and measured to validate the design concept. Measured results for the prototype are discussed in the experimental validation section. The results indicate an impedance bandwidth of 3080 MHz (3.07 GHz to 6.15 GHz, determined from the 10-dB reflection) with a centre frequency of 3.50 GHz. Moreover, an average peak gain of 5.22 dBi, an average radiation efficiency of 92.58%, and a stable radiation pattern are achieved in the entire operating bandwidth.

## 2. Antenna Design Architecture

**Figure 1:**
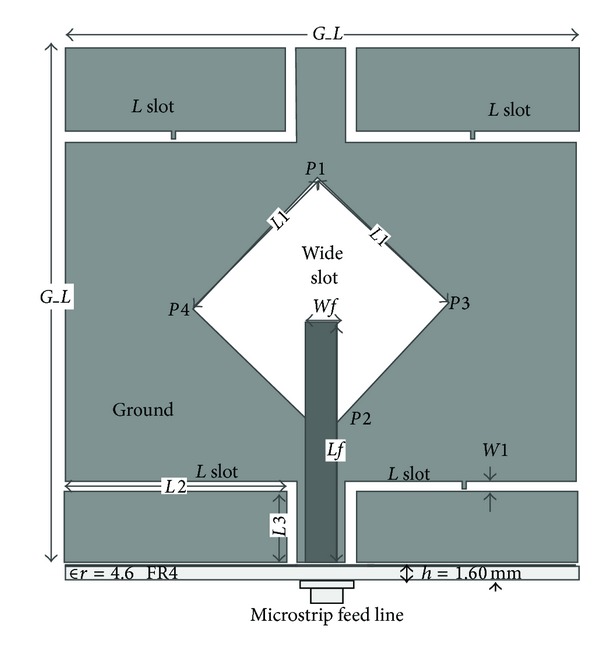
Proposed antenna geometry.

The geometry of the proposed wide-slot defected ground structure antenna is portrayed in Figure 1. The antenna consists of simple wide square slot in the centre of one side of the substrate and fed line is printed on the other side for exciting two modes with close resonance frequency. The rotated square slot has a side length of *L*1 which determines the lower resonant frequency. To decrease the length of *L*1, the lower resonant frequency is shifted upward. Thus, the lower edge of the operating frequency band also goes upward. This is because the decrease in length *L*1 will shorten the effective current path. Therefore, the centre of the rotated square slot is embedded in the middle of the ground plane and up and down diagonal points are in the middle of the I shaped strip line for obtaining a stable symmetric radiation pattern. The proposed antenna is printed on polymer resin substrate FR4 of thickness 1.6 mm and relative permittivity 4.6 with loss tangent 0.02. The rotated square slot and four *L* slots are printed on one side of the substrate and fed line on the other side of the substrate. Ground plane length is denoted by *G*_*L*; *L* slot width is denoted by *W*1, length *L*2,  *L*3, and diagonal point's *P*1, *P*2, *P*3, and *P*4. Microstrip line width and length are denoted by *Wf* and *Lf*. Comparing to the designed antenna in [22], the proposed antenna has better bandwidth, gain, and smaller size. The details of the optimized design parameter are summarized in Table 1.

## 3. Antenna Performance with Epoxy Resin Polymer Substrate

**Figure 2:**
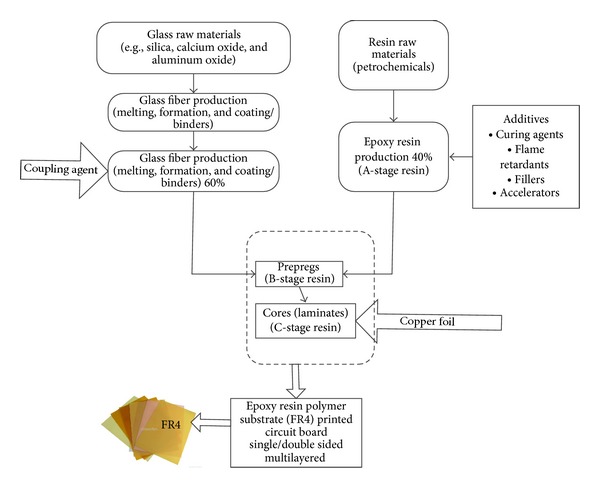
FR4 material construction.

The proposed planar microstrip patch antenna was designed and analyzed using a finite element method- (FEM-) based high-frequency full-wave electromagnetic simulator (HFSS) from the Ansys Corporation. The designed antenna was fabricated on a recently available 1.6 mm thick low-cost durable polymer resin substrate using an in-house printed circuit board (PCB) prototyping machine. The substrate material consists of an epoxy matrix reinforced by woven glass. This composition of epoxy resin and fibre glass varies in thickness and is direction dependent. One of the attractive properties of polymer resin composites is that they can be shaped and reshaped repeatedly without losing their material properties [19]. Due to lower manufacturing cost, ease of fabrication, design flexibility, and market availability of the proposed material, it has become popular for use as a substrate in patch antenna design. The composition ratio of the material is 60% fibre glass and 40% epoxy resin.  Figure 2 shows the several steps involved to construct the epoxy resin polymer substrate (FR4) material substrate.

Glass raw materials are melted in a furnace and extruded to form fibreglass filaments that are combined into strands of multiple fibre yarn. The yarns are then weaved to form fibreglass cloth. A coupling agent, typically an organosilane, is coated onto the fabric to improve the adhesion between organic resin and inorganic glass. Resin is obtained from processing the petrochemicals and in its pure (uncured) form is called A-stage resin. Additives such as curing agents, flame retardants, fillers, and accelerators are added to the resin to tailor the performance of the board. A prepreg is fabricated from a 60% glass fabric impregnated with the semicured (B stage) 40% epoxy resin. Multiple prepregs are thermally pressed to obtain a core or laminate (C-stage resin). Copper foil is then typically electrodeposited to obtain a copper clad laminate. Thus, a final product of FR4 material substrate has come to market.

**Figure 3:**
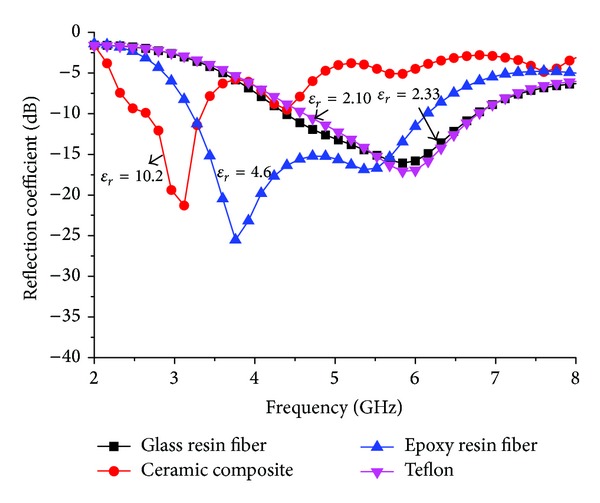
Effect of reflection coefficient for four different dielectric materials.

Figure 3 shows the effect of the different substrate materials on the return loss of the proposed antenna. It can be clearly seen that the proposed antenna provides a wider bandwidth and acceptable return loss value compared with the three other reported materials. The dielectric constant and loss tangent of epoxy resin fibre is comparatively low so bandwidth is increased. Although the antenna with a ceramic-PTFE composite material substrate gives a lower frequency return loss value because of the higher dielectric, the desired resonances are shifted and it is extremely expensive compared with the proposed material.  Table 2 shows the dielectric properties and achieved bandwidth from the proposed design with different materials.

**Figure 4:**
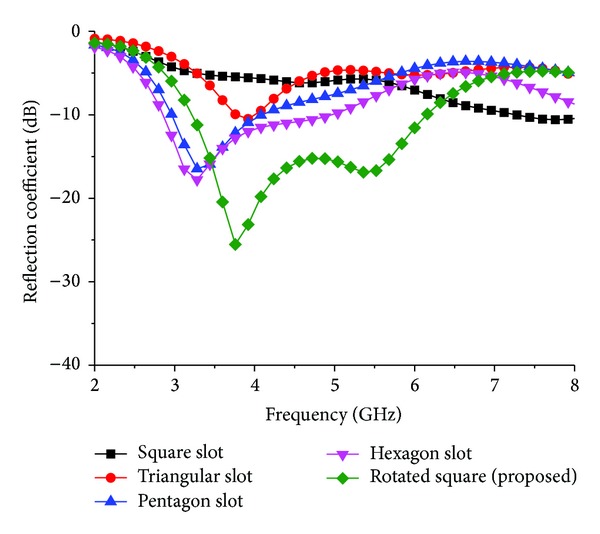
Effect of reflection coefficient for different slot shapes.

**Figure 5:**
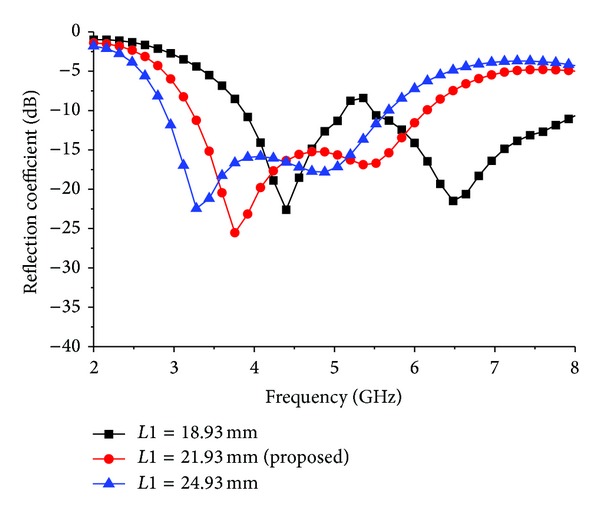
Effect of reflection coefficient for different values of *L*1.

Figure 4 depicts the reflection coefficient of the different types of slots in the ground plane. By using the square slot, there is no resonance. There is a little resonance in that operating band for the triangular slot. However, a better operating band is achieved by using the pentagon and hexagon slots. The maximum bandwidth is achieved by etching the rotating square slot with diagonal points *P*1 and *P*2 in the middle of the strip line.  Figure 5 shows the simulated reflection coefficient of the proposed antenna for different values of *L*1. The other parameter values used in this simulation remain unchanged. It can be seen that resonance frequencies are shifted upward when *L*1 increases and downward when *L*1 increases. Finally, it can be observed that the simulated input impedance has the widest value (3000 MHz) at the length of *L*1 = 21.93 mm.

**Figure 6:**
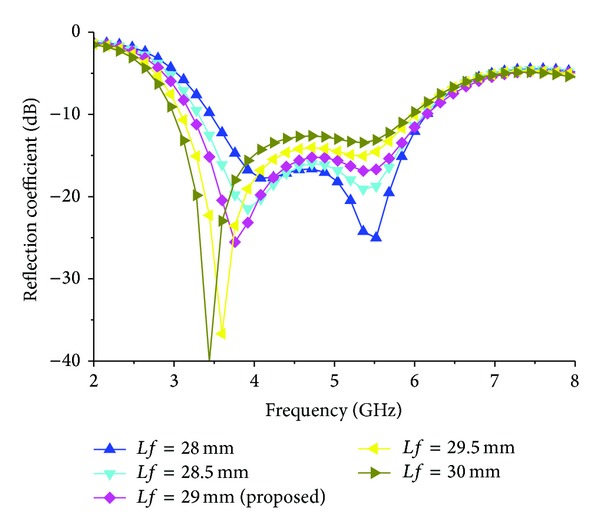
Effect of reflection coefficient for different values of *Lf*.

**Figure 7:**
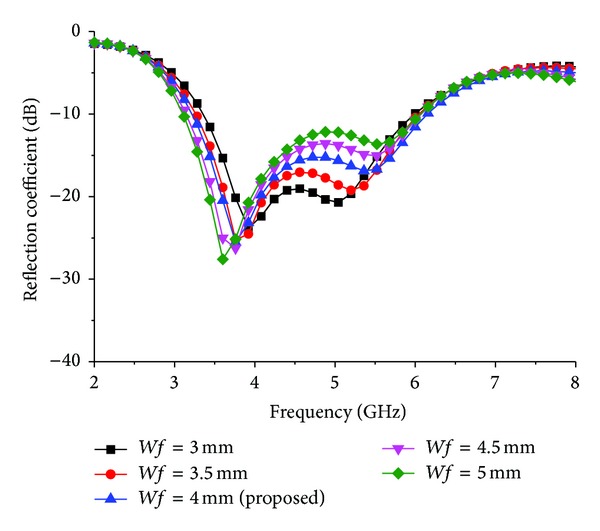
Effect of reflection coefficient for different feed width *Wf*.

An important feature of the proposed antenna design is the stimulus of impedance matching caused by the coupling effects between wide slot and feed length and width. For this reason, the effect of the length *Lf* = 28, 28.5, 29, 29.5, and 30 mm on the performance of the proposed design is investigated and depicted in Figure 6. The other parameter remains unchanged as in Table 1. Due to the variety of *Lf*, both lower and upper resonances have large changes. This is because increasing the length of *Lf* significantly increases the total capacitive effect and thus lowers the lowest resonance frequency while decreasing the operating band. The impedance bandwidth changes significantly for variation of *Lf* because of the sensitivity of the impedance matching to this parameter. However, with the decrease in the length of *Lf*, the upper resonance shifts upward and impedance bandwidth decreases. With the length of *Lf* chosen to be 29.50 mm, the impedance bandwidth has the widest value in this investigation. The width of the strip line has a minor effect on the lower resonant mode but a large effect on the upper resonant mode, as shown in Figure 7. The optimized value of *Wf* is 4 mm where widest impedance bandwidth is achieved.

**Figure 8:**
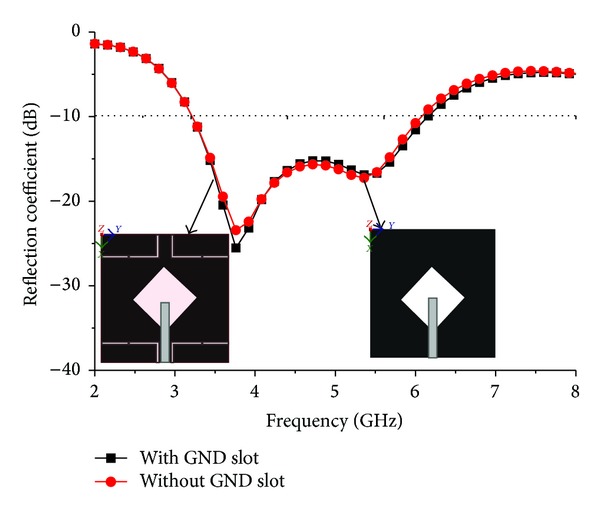
Effect of reflection coefficient with and without slot in ground plane.

**Figure 9:**
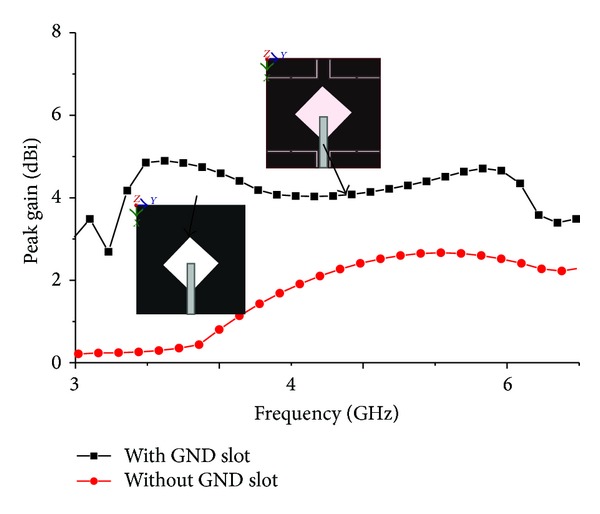
Effect of peak gain with and without slot in ground plane.

**Figure 10:**
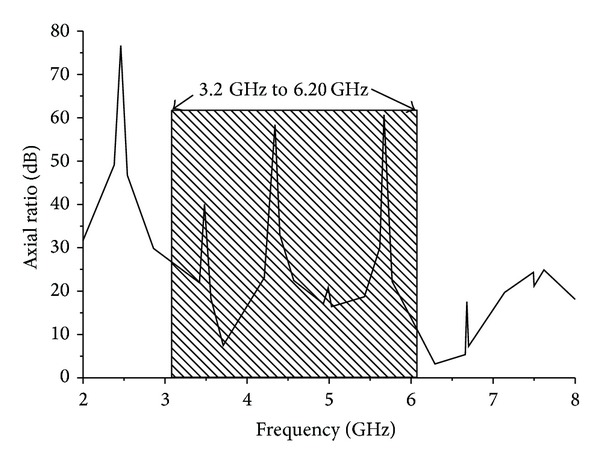
Axial ratio of the proposed antenna.

**Figure 11:**
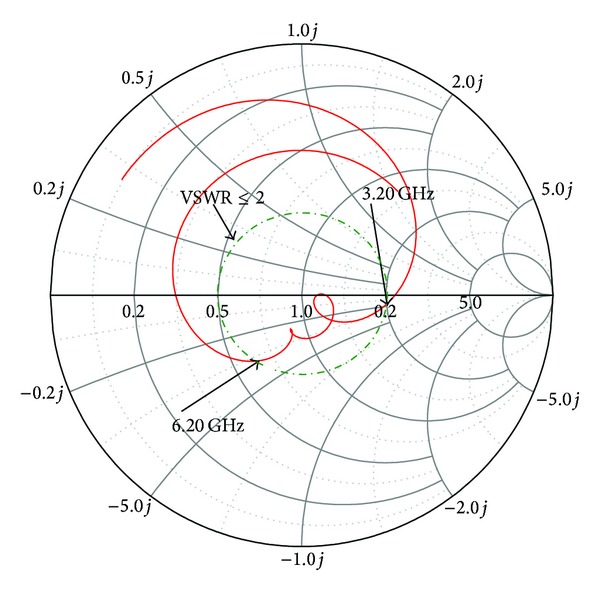
Smith chart of the proposed antenna.

Figures 8 and 9 show the effect of reflection coefficient and achieved peak gains of the proposed antenna with and without the use of an *L* slot in the ground plane. It can be clearly seen that there is a minor effect in the reflection coefficient but a major improvement in terms of gain. The insertion of *L*-shaped slots in the ground plane created some sort of discontinuity which caused the electric current launched by the primary radiator to reroute its path along the conducting surface of the ground. As a result, the electrical length of the ground is increased. With the strong coupling from the radiator, the ground slots cause a considerable impact on the input impedance. This positive coupling effect is responsible for increasing gain.  Figure 10 shows the axial ratio of the proposed antenna. Generally, the axial ratio is considered to determine antenna polarization. Antennas are circularly polarized if the value of the axial ratio is less than 3 dB. For an ideal circularly polarized antenna the axial ratio is 0 dB. It can be clearly seen that the axial ratio is larger than 3 dB, which means the proposed antenna is linearly polarized. At the six resonant frequencies of 3.20 GHz, 3.50 GHz, 3.70 GHz, 5.2 GHz, 5.5 GHz, and 5.8 GHz, the axial ratio values are 22.68 dB, 30.30 dB, 8.06 dB, 16.30 dB, 20.77 dB, and 19.68 dB, respectively. It can be understood that the axial ratio decreases with higher frequency due to the low current intensity in the upper side of the patch and opposite direction. The input impedance and the voltage standing wave ratio are validated in the Smith chart shown in Figure 11. Three of the resonances are in the 2 : 1 VSWR circle, and input impedance is close to the standard 50 Ohm. The Rx values in the Smith chart table represent the input impedance. The curve has a tight resonant loop close to the centre of the Smith chart. This means that the proposed antenna greatly enhances the impedance bandwidth. The markers m1 and m2 represent the start and ending frequencies of the operating band.

**Figure 12:**
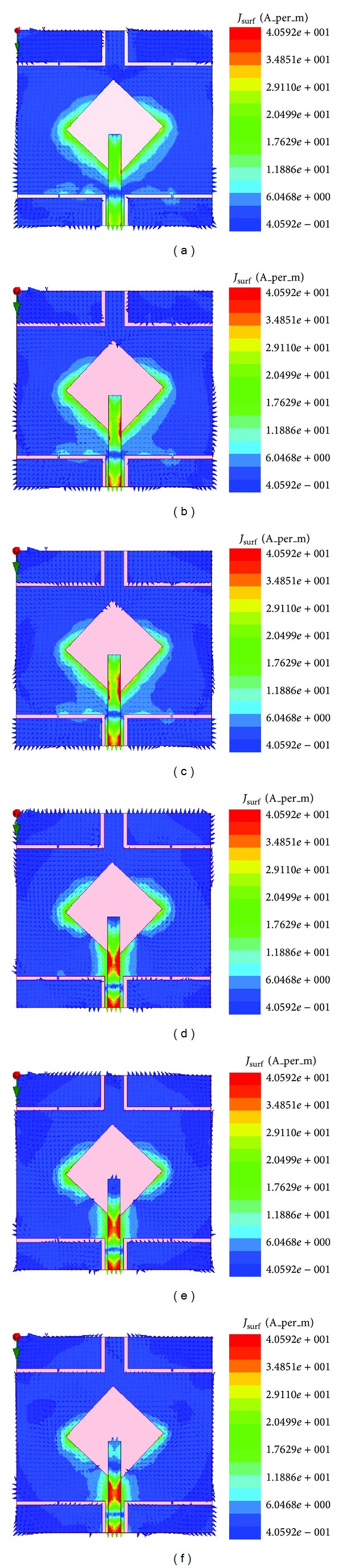
Surface current distribution of the proposed antenna at (a) 3.20 GHz, (b) 3.50 GHz, (c) 3.70 GHz, (d) 5.2 GHz, (e) 5.5 GHz, and (f) 5.8 GHz.

Figure 12 shows the surface current distribution of the radiating patch element of the proposed antenna at 3.20 GHz, 3.50 GHz, 3.70 GHz, 5.2 GHz, 5.5 GHz, and 5.8 GHz, respectively. It has been observed that at the lower band the current intensity is much weaker. The current is more exciting in strip line and wide slotted diagonal points. Specially, the left and right arms of wide slot are more excited. Besides, *L* slots in ground plane are also more excited than plane area. Therefore, from the relationship between gain, power and current of the proposed antenna can be validated from the current distribution.

**Figure 13:**
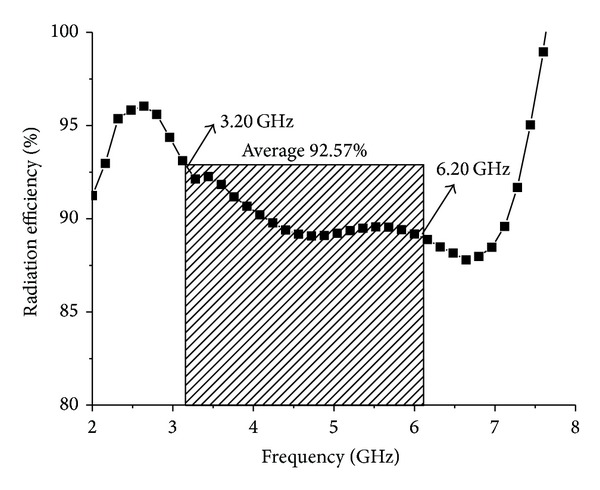
Radiation efficiency of the proposed antenna.

Figure 13 shows the radiation efficiency of the proposed antenna. It can be seen that 92.58% of average radiation efficiency is achieved in the entire operating band. The radiation efficiency at the lower resonant frequencies of 3.20 GHz, 3.50 GHz, and 3.70 GHz is achieved at 92.58%, 92.04%, and 91.41%, respectively. On the other hand, at the upper resonant frequencies of 5.2 GHz, 5.5 GHz, and 5.8 GHz, the radiation efficiencies are 89.35%, 89.56%, and 89.45%, respectively.

## 4. Experimental Verification

**Figure 14:**
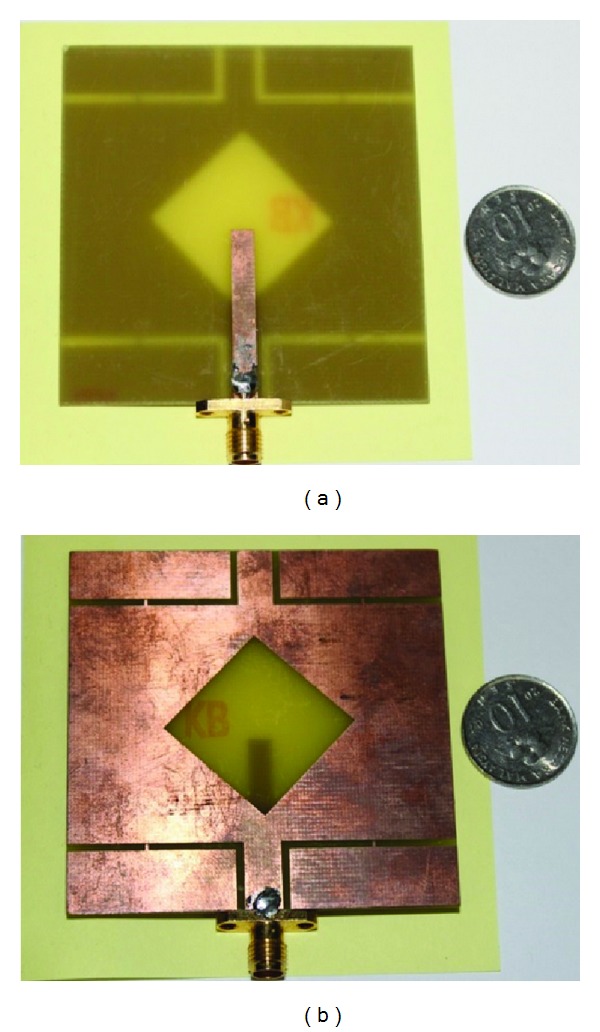
Fabricated prototype: (a) front view, (b) back view.

**Figure 15:**
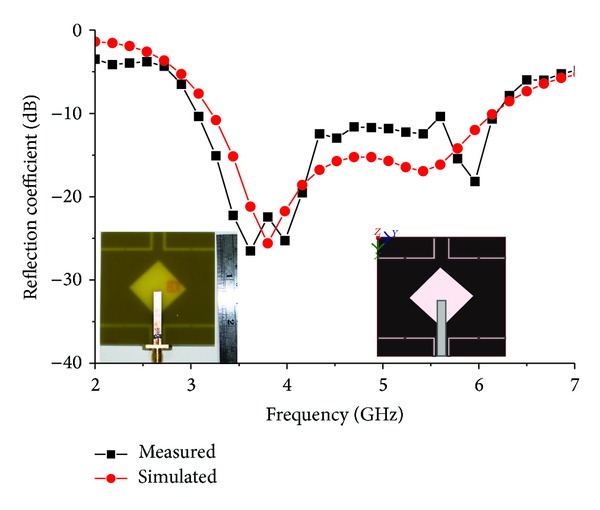
Comparison between simulated and measured reflection coefficient of the proposed antenna.

**Figure 16:**
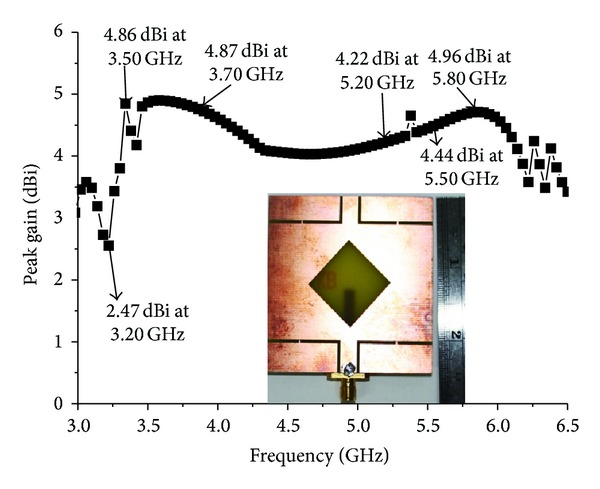
Measured gain of the proposed antenna.

The performance of the proposed antenna was analysed and optimized using a FEM-based high-frequency 3D full-wave electromagnetic simulator, Ansoft's HFSS, and plotted using the scientific graphing and data analysis software OriginPro and Excel. The results of the proposed antenna prototype were measured in a rectangular-shaped 5.5 m × 5 m × 3.5 m anechoic measurement chamber. A double ridge guide horn antenna was used as a reference antenna. The high-power broadband honeycomb pyramidal-shaped electrically thick foam absorber with less than −60 dB reflectivity at normal incidence was used on the wall, ceiling, and floor. A turn table with a diameter of 1.2 m was used to rotate the measuring antenna with the following specifications: 1 rpm rotation speed; 360° rotation angle connected with a 10-meter cable between controllers. An Agilent vector network analyser (VNA) with a range of up to 20 GHz was used for the measurement procedure.  Figure 14 shows a photograph of the proposed antenna prototype.  Figure 15 shows the simulated and measured return loss of the optimized proposed antenna. A slight discrepancy occurred, which led to the differences between simulated and measured return loss value due to the effect from soldering of the SMA (Sub Miniature Version A) connector and the loss from the connecting cable. The results show that the antenna provides a very wide impedance bandwidth of over 88.07% from a frequency of 3.07 to 6.15 GHz for which S11 <−10 dB. Compared with the antenna proposed in [18], the impedance bandwidth is significantly wider by more than 1000 MHz. Detailed numerical and experimental investigations confirm that the achieved impedance bandwidth is limited by the impedance match between the microstrip line height and width, the rotated square slot diagonal points with respect to feed, and the wide square slot arm's length.  Figure 16 shows the measured gain of the proposed antenna. The highest peak gain of the proposed antenna is 4.87 dBi at 3.70 GHz and lowest 2.47 dBi at 3.20 GHz. The average peak gain of the proposed antenna is 4.17 dBi in the entire operating band. This type of antenna gain is good for WLAN/WiMAX applications.

**Figure 17:**
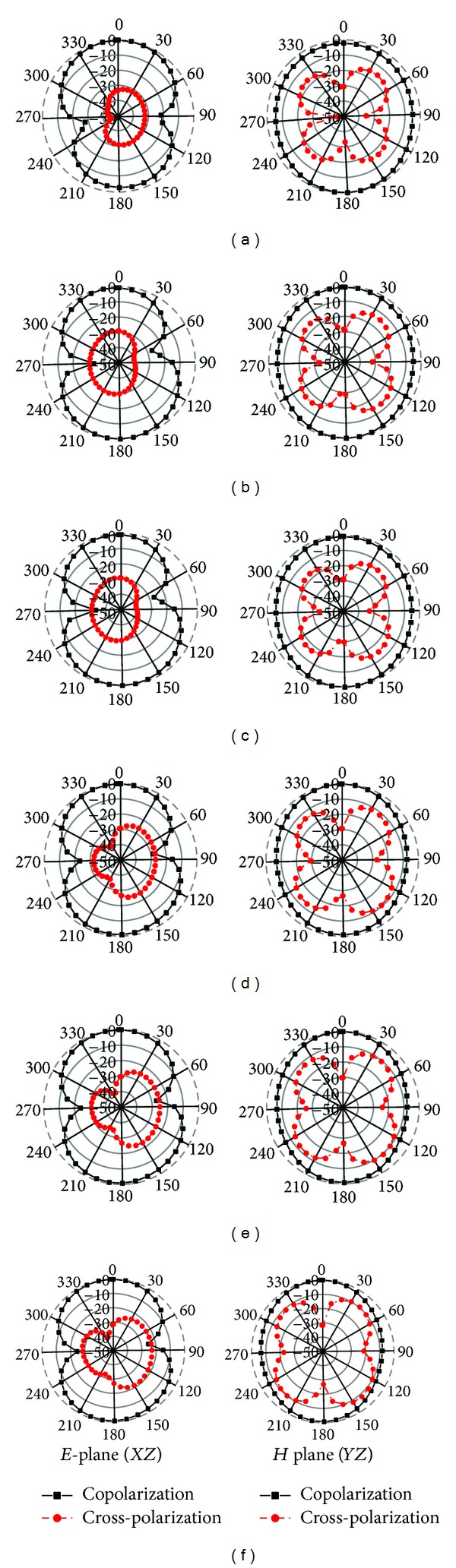
Radiation pattern of the proposed antenna at (a) 3.20 GHz, (b) 3.50 GHz, (c) 3.70 GHz, (d) 5.2 GHz, (e) 5.5 GHz, and (f) 5.8 GHz.

Figure 17 shows the measured *E(XZ)-H(YZ)* plane normalized radiation pattern of the proposed antenna prototype at different frequencies. It can be undoubtedly seen that good omnidirectional characteristics are obtained for the proposed antenna excited at all other frequencies across the operating band. Furthermore, the effect of cross-polarization is much smaller than the copolarization desired. Since the proposed antenna design structure is symmetrical, the radiation patterns are also in symmetry with respect to the antenna axis (*θ* = 0°). Although at higher frequencies, more harmonics are observed mainly in the cross-polarization radiation field, the antenna has a good stable radiation without gain degradation.

Table 3 compares the proposed and some existing antennas. The table shows that the proposed antenna achieves wider bandwidth and higher gain with smaller size compared with the reported antennas, although some of the reported antennas obtain a wide bandwidth and higher gain compromising the overall size and structure.

## 5. Conclusion

A printed modified wide-square slot fed by a 50 *Ω* microstrip line with slot diagonal points embedded on the middle of the strip line is presented in this paper. By introducing rotated square slot diagonal points in the middle of the strip line, the impedance bandwidth of the proposed wide-slot antenna can be significantly enhanced. In addition, the size of the proposed antenna can be reduced. Moreover, the four *L*-shaped slots are embedded in the ground to increase the gain of the antenna. With the optimized antenna geometry, the proposed antenna offers a measured impedance bandwidth over 88%. The proposed structure reveals an average peak gain of 4.17 dBi, above 92.58% average radiation efficiency, stable far-field radiation characteristics, and low cross-polarization in the entire operating bandwidth. By properly choosing the suitable slot shape position and tuning their dimensions parameter with simulation software, the proposed design with wide operating bandwidth, relative small size, peak gain, and improved radiation pattern is obtained. Therefore, the proposed antenna is feasible for use as a low-profile, low-cost wideband antenna for WLAN/Wi-MAX applications.

## Figures and Tables

**Table 1:** Proposed antenna specification.

Parameter	(mm)	Parameter	(mm)
*G*_*L*	62.00	*Wf*	4
*W*1	0.50	*Lf*	29.50
*L*1	22.93	*h*	1.60
*L*2	27.00	*L*3	10.00

**Table 2:** Dielectric properties of substrate materials.

Substrate material	Relative permittivity (*ϵ*_*r*_)	Dielectric loss tangent	Fraction achieved bandwidth (%)	Antenna dimension
Glass microfiber reinforced PTFE	2.33	0.0012	40.44369	As Table 1 parameter
Epoxy resin-fibre glass	4.60	0.02	85.71429	
PTFE ceramic	10.20	0.0023	21.63934	
Teflon	2.10	0.01	36.61017	

**Table 3:** Comparison between proposed and some existing antennas.

Reference	Dimension (mm)	Bandwidth (MHz)	Peak gain
[22]	70 × 70	2200 (49.40%)	5.7 dBi
[17]	53.7 × 53.7	1091 (57.42%)	4.5 dBi
[23]	80 × 80	3510 (118.4%)	4.6 dBi
Proposed	62 × 62	3000 (88.07%)	4.17 dBi

## References

[1] Balanis CA (2012). *Antenna Theory: Analysis and Design*.

[2] Oohira K (2008). Development of an antenna material based on rubber that has flexibility and high impact resistance. *Technical Review*.

[3] Art Aguayo S (2010). Analyzing advances in antenna Materials. *Antenna SyStems and Technology*.

[4] Ullah MH, Islam MT, Mandeep J, Misran N (2013). Ceramic-polytetrafluoroethylene composite material-based miniaturized split-ring patch antenna. *Science and Engineering of Composite Materials*.

[5] Ullah MH, Islam MT (2013). Miniaturized modified circular patch monopole antenna on ceramic-polytetrafluroethylene composite material substrate. *Journal of Computational Electronics*.

[6] Assis RRd, Bianchi I (2012). Analysis of microstrip antennas on carbon fiber composite material. *Journal of Microwaves, Optoelectronics and Electromagnetic Applications*.

[7] Samsuzzaman M, Islam MT, Mandeep JS (2013). Parametric analysis of a glass-micro fibre-reinforced PTFE material, multiband, patch-structure antenna for satellite applications. *Optoelectronics and Advanced Materials*.

[8] Ullah MH, Islam MT (2013). A compact square loop patch antenna on high dielectric ceramic—PTFE composite material. *Applied Physics A*.

[9] Rajgopal SK, Sharma SK (2009). Investigations on ultrawideband pentagon shape microstrip slot antenna for wireless communications. *IEEE Transactions on Antennas and Propagation*.

[10] Ullah MH, Islam MT, Mandeep JS, Misran N (2013). A new double L-shaped multiband patch antenna on a polymer resin material substrate. *Applied Physics A*.

[11] Kharakhili FG, Fardis M, Dadashzadeh G, Ahmadi A, Hojjat N (2007). Circular slot with a novel circular microstrip open ended microstrip feed for UWB applications. *Progress in Electromagnetics Research*.

[12] Li P, Liang J, Chen X (2006). Study of printed elliptical/circular slot antennas for ultrawideband applications. *IEEE Transactions on Antennas and Propagation*.

[13] Liu Y, Lau K, Xue Q, Chan C (2004). Experimental studies of printed wide-slot antenna for wide-band applications. *IEEE Antennas and Wireless Propagation Letters*.

[14] Li Y, Li W, Ye Q (2013). A CPW-fed circular wide-slot UWB antenna with wide tunable and flexible reconfigurable dual notch bands. *The Scientific World Journal*.

[15] Mobashsher AT, Islam MT, Misran N (2011). Wideband compact antenna with partially radiating coplanar ground plane. *Applied Computational Electromagnetics Society Newsletter*.

[16] Azim R, Islam MT, Misran N (2010). Printed planar antenna for wideband applications. *Journal of Infrared, Millimeter, and Terahertz Waves*.

[17] Sze J-Y, Wong K-L (2001). Bandwidth enhancement of a microstrip-line-fed printed wide-slot antenna. *IEEE Transactions on Antennas and Propagation*.

[18] Dissanayake T, Esselle KP (2008). UWB performance of compact L-shaped wide slot antennas. *IEEE Transactions on Antennas and Propagation*.

[19] Sung Y (2012). Bandwidth enhancement of a microstrip line-fed printed wide-slot antenna with a parasitic center patch. *IEEE Transactions on Antennas and Propagation*.

[20] Chen WL, Wang GM, Zhang CX (2009). Bandwidth enhancement of a microstrip-line-fed printed wide-slot antenna with a fractal-shaped slot. *IEEE Transactions on Antennas and Propagation*.

[21] Krishna D, Gopikrishna M, Aanandan CK, Mohanan P, Vasudevan K (2009). Compact wideband Koch fractal printed slot antenna. *IET Microwaves, Antennas and Propagation*.

[22] Jan JY, Su JW (2005). Bandwidth enhancement of a printed wide-slot antenna with a rotated slot. *IEEE Transactions on Antennas and Propagation*.

[23] Sung Y (2011). A printed wide-slot antenna with a modified l-shaped microstrip line for wideband applications. *IEEE Transactions on Antennas and Propagation*.

